# The host restriction factor SERINC5 inhibits HIV-1 transcription by negatively regulating NF-κB signaling

**DOI:** 10.1016/j.jbc.2024.108058

**Published:** 2024-12-07

**Authors:** Weiting Li, Meng Qu, Tianxin Zhang, Guoqing Li, Ruihong Wang, Yinghui Tian, Jialin Wang, Bin Yu, Jiaxin Wu, Chu Wang, Xianghui Yu

**Affiliations:** 1 State Key Laboratory for Diagnosis and Treatment of Severe Zoonotic Infectious Diseases/Key Laboratory for Zoonosis Research of the Ministry of Education, School of Life Sciences, Jilin University, Changchun, China; 2 National Engineering Laboratory for AIDS Vaccine, School of Life Sciences, Jilin University, Changchun, China; 3 Key Laboratory for Molecular Enzymology and Engineering, the Ministry of Education, School of Life Sciences, Jilin University, Changchun, China

**Keywords:** HIV-1, NF-κB signaling pathway, MDA5, RIG-I, SERINC5, transcription

## Abstract

Serine incorporator 5 (SER5) can be incorporated into HIV-1 virions to block viral entry by disrupting the envelope glycoprotein-mediated viral fusion to the plasma membrane. Recent studies suggest that SER5 also inhibits HIV-1 mRNA transcription and the subsequent progeny virion biogenesis. However, the underlying mechanisms through which SER5 antagonizes the viral transcription remain poorly understood. Here, we demonstrate that SER5 inhibits HIV-1 transcription by negatively regulating NF-κB signaling, which is mediated by the retinoic acid-inducible gene I-like receptors, MDA5 and RIG-I. By recruiting TRIM40 as the E3 ubiquitination ligase to promote K48-linked polyubiquitination and proteasomal degradation of MDA5 and RIG-I, SER5 impedes nuclear translocation of the p50/p65 dimer, resulting in repression of HIV-1 LTR-driven gene expression. Hence, our findings strongly support a role for SER5 in restricting HIV-1 replication through inhibition of NF-κB-mediated viral gene expression.

The human serine incorporator (SERINC) family proteins, consisting of five members (SERINC1 to SERINC5), are multipass transmembrane proteins that have been identified as host restriction factors against human immunodeficiency virus (HIV) (1, 2, 3, 4). Among these proteins, SERINC5 (SER5) and, to a lesser extent, SERINC3 (SER3), potently inhibit viral infectivity at the entry step by being incorporated into budding viral particles (2, 3, 5). In contrast, SERINC1 (SER1) displays limited or no activity against HIV-1 (2, 6). Besides the effect on HIV, SER5 also shows antiviral activity against a variety of retroviruses including simian immunodeficiency virus (SIV), murine leukemia virus (MLV), and equine infectious anemia virus (EIAV), as well as other enveloped viruses such as hepatitis B virus, classical swine fever virus (CSFV), SARS-CoV-2, and influenza A virus (2, 7, 8, 9, 10, 11, 12). To counteract this antiviral mechanism, the Nef protein encoded by HIV-1 downregulates the expression of SER5 on the cell surface through lysosomal degradation (2, 3, 13, 14). Additionally, SIV Nef, MLV glycosylated Gag, EIAV accessory protein S2, and SARS-CoV-2 ORF7 also exert antagonistic effects on the antiviral activity mediated by SER5 (2, 8, 12, 15, 16).

Until now, the antiviral mechanism of SER5 remains incompletely elucidated. Recent investigations have revealed that SER5 exerts its inhibitory effect on HIV-1 fusion pore formation by promoting functional inactivation of the envelope (Env) glycoprotein (17, 18). Furthermore, it has been suggested that the direct interaction between Env and SER5 may induce conformational changes in Env, leading to impairment of its function (19, 20, 21, 22). Apart from impacting Env, recent studies have demonstrated that SER5 also influences viral mRNA synthesis and subsequently impedes progeny virion biogenesis (23, 24), thereby indicating its involvement in multiple stages of the viral life cycle. However, the underlying mechanisms through which SER5 antagonizes the viral transcription remain poorly understood. Despite not being induced by interferons (IFNs) like other classical antiviral restriction factors, it appears that SER5 plays a positive role in regulating innate immune signaling by promoting increased production of type I IFNs (IFN-I) and pro-inflammatory cytokines, thereby restricting the infections caused by HIV-1, vesicular stomatitis virus, Zika virus, and CSFV (10, 25).

Innate immune responses to viral infection are mediated by pattern recognition receptors and a variety of ligands, such as cytokines (26). Retinoic acid-inducible gene I (RIG-I)-like receptors (RLRs), including RIG-I and melanoma differentiation-associated gene-5 (MDA5), can recognize viral RNA (27, 28). Upon ligand binding, RIG-I and MDA5 activate the adaptor protein mitochondrial antiviral signaling (MAVS) (29), which further recruits tumor necrosis factor (TNF) receptor-associated factors (TRAF2, TRAF5, and TRAF6) to activate the IκB kinase (IKK) complex (30). The IFN-I and nuclear factor-κB (NF-κB) signaling pathways are activated through diverse IKKs-mediated signal transduction involving TBK1/IKKε and IKKα/β/γ (IKKγ is also known as NEMO) (31, 32). This leads to the nuclear translocation of IRF3/IRF7 and p50/p65 (NF-κB1/RelA) heterodimers, respectively, which is required for the expression of IFN-I (including IFN-α and IFN-β) as well as pro-inflammatory cytokines (33, 34, 35, 36). In addition to playing a role in inducing IFN-β and pro-inflammatory responses, NF-κB signaling is also critical for regulating HIV-1 proviral transcription. Activation of the canonical NF-κB signaling pathway enhances the transcriptional activity of integrated HIV-1 genome because the p50/p65 dimer binds to an NF-κB-responsive element in the transcriptional control region of HIV-1 long terminal repeats (LTRs) in the nucleus (37, 38, 39). Conversely, retention of the p50/p65 dimer in cytoplasm inhibits early transcription levels of HIV-1. Therefore, factors that inhibit the NF-κB signaling pathway can reduce the transcriptional activity of HIV-1.

Here, we demonstrate that the mechanism of SER5-mediated HIV-1 mRNA transcription block is exhibited by negatively regulating the RLR-induced NF-κB signaling during viral replication. By recruiting TRIM40 as the E3 ubiquitination ligase, SER5 mediates the proteasomal degradation of MDA5 and RIG-I, subsequently inhibiting the nuclear translocation of the p50/p65 dimer and its binding to HIV-1 LTR.

## Results

### SER5 reduces HIV-1 virion production by downregulating viral transcription

**Figure 1:**
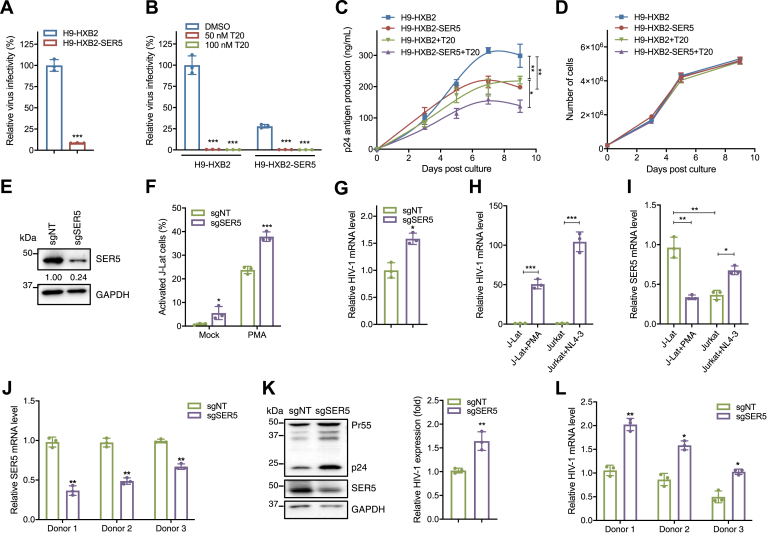
**SER5 inhibits HIV-1 production by downregulating viral gene expression at the mRNA level.***A*, H9-HXB2 and H9-HXB2-SER5 cells were cultured for 48 h, and the infectivity of equal virions (normalized by the level of the p24 protein) in the supernatants was tested in TZM-bl cells. *B*, equal amounts of the viruses produced from H9-HXB2 and H9-HXB2-SER5 cells and different concentrations of T20 were added into TZM-bl cells for incubation of 48 h to detect the viral infectivity. *C* and *D*, H9-HXB2 and H9-HXB2-SER5 cells were cultured with 50 nM T20 or DMSO for 9 days. T20 was added to the culture medium once a day. Viral production (*C*) was monitored at the indicated time points and the number of living cells (*D*) was determined. *E*–*G*, J-Lat 6.3 cells were infected with CRISPR lentiviruses carrying Cas9-sgRNA targeting SER5 (sgSER5) or a random negative target (sgNT), and the expression of SER5 was analyzed by Western blotting (*E*). Then, the activated GFP-positive cells of J-Lat-sgNT and J-Lat-sgSER5 cells stimulated with PMA (50 ng/ml) for 48 h were detected by flow cytometry (*F*). The mRNA levels of HIV-1 Gag in PMA-activated J-Lat-sgNT and J-Lat-sgSER5 cells were analyzed by RT-qPCR (*G*). *H* and *I*, J-Lat 6.3 cells were stimulated with PMA for 48 h and Jurkat cells were infected with NL4-3 for 72 h. The mRNA levels of Gag (*H*) and SER5 (*I*) were analyzed by RT-qPCR. *J*–*L*, PBMCs isolated from the blood of three healthy donors were activated with 5 μg/ml phytohemagglutinin (PHA) for 72 h, infected with the sgSER5 or sgNT lentivirus for 48 h, and then selected by puromycin for another 3 days, followed by infection of these cells with VSV-G-pseudotyped NL4-3 for 48 h. The mRNA levels of SER5 (*J*) and Gag (*L*) were analyzed by RT-qPCR. The expression of viral proteins in the cell lysates (*K*) was analyzed by Western blotting (*left* panel) and the difference of the p24 levels between the sgSER5 and sgNT groups (*right* panel) was analyzed. In (*A*–*D*) and (*F*–*L*), data are represented from three independent experiments. In (*K*), the viral p24 expression levels were calculated relative to the corresponding GAPDH levels. In (*A*), (*B*), (*F*–*L*), ∗, *p* < 0.05; ∗∗, *p* < 0.01; ∗∗∗, *p* < 0.001 (unpaired Student’s *t* test). In (*C*), ∗, *p* < 0.05; ∗∗, *p* < 0.01 (repeated-measured ANOVA).

To investigate the regulatory role of SER5 in HIV-1-infected T lymphocytes, green fluorescent protein (GFP)-fused SER5 was transduced into H9 cells chronically infected by an HIV-1 strain HXB2 (H9-HXB2) by lentiviral infection. The positive rate of stable SER5 expression in the transduced H9-HXB2 cells (H9-HXB2-SER5) was detected by flow cytometry analysis (Fig. S1*A*). The culture supernatants from H9-HXB2 and H9-HXB2-SER5 cells were harvested and equal virions (normalized by the level of the p24 protein) were used to infect TZM-bl reporter cells (40). As expected, high expression of SER5 in H9-HXB2 cells reduced the virus infectivity (Fig. 1*A*). To eliminate the potential antiviral effect caused by virion-incorporated SER5 on intercellular viral spreading, Enfuvirtide (T20) was used to block HIV-1 fusion at the cell membrane (41, 42, 43). Virus supernatants collected from H9-HXB2 and H9-HXB2-SER5 cells were mixed with different concentrations of T20 for infection assays using TZM-bl cells. We observed complete inhibition of viral infection with 50 nM T20 concentration (Fig. 1*B*). Next, the impact of SER5 on virion production from H9-HXB2 cells was evaluated in the presence or absence of T20 treatment. The results showed that both high expression levels of SER5 and addition of T20 significantly reduced the viral production from H9-HXB2 cells (Fig. 1*C*), without affecting the cell growth rates (Fig. 1*D*). Surprisingly, in the presence of T20, the viral production from H9-HXB2-SER5 cells remained significantly lower than that from H9-HXB2 cells (Fig. 1*C*), indicating that SER5 not only inhibits the viral entry into cells but also reduces HIV-1 virion production from infected cells.

To investigate the potential suppression of HIV-1 gene expression by endogenous SER5, we knocked down the level of SER5 in chronically infected Jurkat T cells (J-Lat 6.3 cells) harboring a full-length HIV-1 provirus with a GFP gene, thus providing a cellular model for studying HIV-1 latency. J-Lat 6.3 cells were transduced with CRISPR lentiviruses carrying Cas9-sgRNA targeting SER5 and the resulting SER5 depletion was confirmed by Western blotting (Fig. 1*E*). Upon PMA stimulation, the SER5-knockout cells exhibited reactivation of latent HIV-1 accompanied by significantly increased LTR-driven GFP reporter expression compared to the control cells as measured by flow cytometry (Fig. 1*F*). Furthermore, knockout of SER5 led to elevated mRNA levels of HIV-1 Gag in J-Lat 6.3 cells upon PMA treatment (Fig. 1*G*), indicating that reducing SER5 expression facilitates reactivation of the latent virus. To determine whether active HIV-1 replication affects the endogenous expression of SER5, we examined the mRNA level of SER5 when J-Lat 6.3 cells were activated using PMA and Jurkat cells were infected with HIV-1 strain NL4-3. Significantly increased levels of HIV-1 mRNA were observed upon PMA stimulation and NL4-3 infection (Fig. 1*H*). Viral infection of Jurkat cells resulted in an upregulation of SER5 mRNA, indicating that SER5 could respond to newly-established HIV-1 infection (Fig. 1*I*). The basal expression level of SER5 in J-Lat 6.3 cells was significantly higher than that in the parental cell line Jurkat, while reactivation of the latent virus by PMA led to a downregulation of SER5 (Fig. 1*I*), suggesting a potential association between physiological SER5 levels and HIV-1 latency status. Moreover, endogenous SER5 was knocked out in human peripheral blood mononuclear cells (PBMCs) obtained from three healthy seronegative donors (Fig. 1*J*), followed by infection of these cells with vesicular stomatitis virus glycoprotein (VSV-G)-pseudotyped NL4-3 carrying a luciferase reporter gene to exclude the antiviral function exerted by SER5 against viral infections (2, 44). Knockout of SER5 in PBMCs resulted in significant increases in the Gag precursor protein Pr55 and capsid p24 expression as well as viral mRNA levels (Fig. 1, *K* and *L*). These results clearly indicate that SER5 impairs progeny virion production from infected cells by reducing the transcription level of HIV-1.

We also compared the activity of SER5 with another HIV-1 restriction factor, BCA2, which has been reported to interfere with early-stage HIV-1 proviral transcription (45). The results demonstrated that SER5 significantly downregulated the viral mRNA levels by 2-fold and 4-fold at 24 h and 48 h post-transfection, respectively, while BCA2 downregulated the viral mRNA levels as early as 10 h post-transfection (Fig. S1*B*). Moreover, we verified the function of SER5 using different coreceptor tropisms and subtypes of HIV-1 proviral molecular clones. Overexpression of SER5 in HEK293T cells resulted in reduced viral protein levels of HIV-1 strain 90CF402 (subtype A/E), ZM247 (subtype C), and JRCSF (subtype B, CCR5 tropism) as that of NL4-3 and HXB2 (subtype B, CXCR4 tropism) (Fig. S1*C*), suggesting a highly conserved role of SER5 in inhibiting HIV-1 gene expression.

### SER5 downregulates NF-κB signaling in the presence of HIV-1

**Figure 2:**
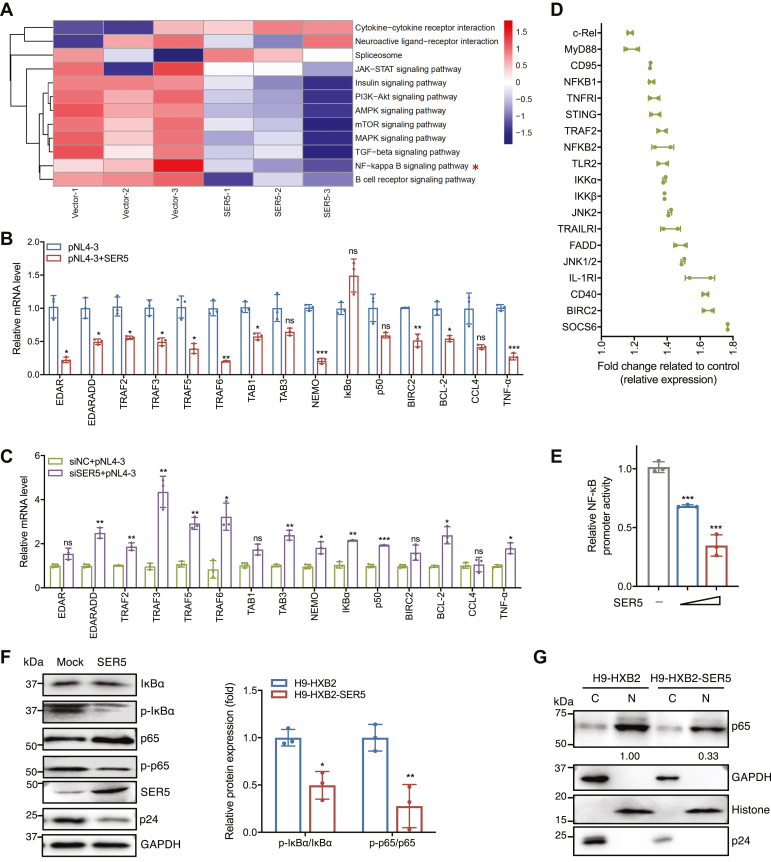
**SER5 suppresses NF-κB signaling and blocks the nuclear translocation of p65.***A*, HEK293T cells were co-transfected with pNL4-3 and SER5 or pVR1012 for 48 h. The heatmap shows the relative gene expression of enriched signaling pathways in SER5-overexpression and control groups using gene set variation analysis (GSVA). *B*, HEK293T cells were co-transfected with pNL4-3 and SER5 or pVR1012 for 48 h. Total RNAs were prepared from the harvested cells and analyzed for the transcriptional levels of the indicated genes by RT-qPCR. *C*, HEK293T cells were transfected with siRNA targeting SER5 (siSER5) or a random sequence as negative control (siNC) and then transfected with pNL4-3 at 24 h post siRNA transfection. The cells were harvested 24 h later and analyzed as described for panel B. *D*, the expression levels of NF-κB signaling pathway-related proteins in HEK293T cells were evaluated using a proteome profiler array kit after 48 h of transfection as described for panel C. Quantitative analysis of the spots was performed *via* densitometry and plotted as fold-changes compared to the internal controls (mean). Data from two technical duplications were analyzed. *E*, 300 ng pNF-κB-luciferase and 50 ng pRenilla-luciferase reporter plasmids were co-transfected with 100 ng or 250 ng of SER5 expression plasmid or pVR1012 into HEK293T cells. The cells were harvested at 36 h post-transfection for assessment of the reporter gene expression by dual luciferase reporter assay. *F*, H9-HXB2 cells with or without stable SER5 expression were cultured with 50 nM T20 for 48 h. The cells were harvested and the endogenous protein levels were then analyzed by Western blotting (*left panel*, one representative result). *Right panel*, the relative expression levels of phosphorylated IκBα (p-IκBα) and p65 (p-p65) calculated relative to the total protein levels of IκBα and p65. *G*, H9-HXB2 cells with or without SER5 stable expression were cultured with 50 nM T20 for 48 h. The cells were harvested for nuclear/cytoplasmic fractionation and then analyzed by Western blotting. p65 levels were calculated relative to the corresponding histone levels. In (*B*), (*C*), (*E*), and (*F*), data are calculated from three independent experiments. ∗, *p* < 0.05; ∗∗, *p* < 0.01; ∗∗∗, *p* < 0.001; ns, not significant (unpaired Student’s *t* test).

To investigate how SER5 inhibits HIV-1 transcription, RNA sequencing (RNA-seq) was performed on HEK293T cells co-transfected with SER5 or its vector and the proviral expression vector of NL4-3 (pNL4-3) which mimics the replication process after HIV-1 genome integration to new virion generation from the infected cells, to enable rapid and deep profiling of transcripts. Based on the differential gene expression analysis, we identified a specific transcriptional regulation feature of SER5 (Fig. S2*A*). Kyoto Encyclopedia of Genes and Genomes (KEGG) analysis showed that the most significantly enriched gene profiles in the upregulation sets induced by SER5 were related to the defense response to virus, regulation of innate immune response, and IFN-I-mediated signaling pathway (Fig. S2*B*), while the downregulation sets were related to the neuroactive ligand-receptor interaction, cytokine-cytokine receptor interaction, and NF-κB signaling pathway (Fig. S2*C*). Interestingly, the NF-κB signaling pathway significantly downregulated by SER5 (Fig. 2*A*) plays an important role in HIV-1 transcription in cells (34, 37, 39). Further analysis revealed that the expression of many genes related to the NF-κB signaling pathway, such as TAB1, TAB3, and TRAF5, were markedly downregulated in the presence of SER5 (Fig. S2*D*). To verify the effect of SER5 on the NF-κB signaling pathway, RNA-seq was repeated on SER5-knockdown cells transfected with pNL4-3. The analysis showed that knockdown of SER5 expression led to significant upregulation of many NF-κB signaling-related genes, such as the TRAF family genes and NFκB1 (Fig. S2*E*). The mRNA levels of the related genes and pro-inflammatory cytokines, such as TNF-α, were further confirmed by reverse transcription-quantitative PCR (RT-qPCR). Consistent with the RNA-seq data, the mRNA levels were significantly decreased or increased following the overexpression or knockdown of SER5 in HEK293T cells, respectively (Fig. 2, *B* and *C*). In addition, a human proteome profiler array for the NF-κB signaling pathway was used to detect whether knockdown of SER5 affected the levels of NF-κB signaling-related proteins in HEK293T cells. The knockdown of SER5 markedly increased the expression levels of many related proteins, including NFκB1, NFκB2, IKKα, IKKβ, and TRAF2, as shown in Figs. 2*D* and S2*F*.

Using a dual luciferase reporter assay, we found that increasing levels of SER5 significantly inhibited NF-κB-mediated luciferase expression (Fig. 2*E*). Moreover, stable expression of SER5 in H9-HXB2 cells led to decreased phosphorylation levels of endogenous IκBα and p65 (Fig. 2*F*). The pivotal event in NF-κB signaling is the translocation of p50/p65 proteins into the nucleus, which facilitates the transcriptional activation of integrated HIV-1 genome (34, 46, 47). By examining the relative abundance of p65 protein in nuclear and cytoplasmic extracts, we found that SER5 partially reduced the nuclear accumulation of p65 in H9-HXB2 cells (Fig. 2*G*). These results suggest that SER5 likely inhibits HIV-1 transcription by downregulating NF-κB-mediated transcriptional activation.

**Figure 3:**
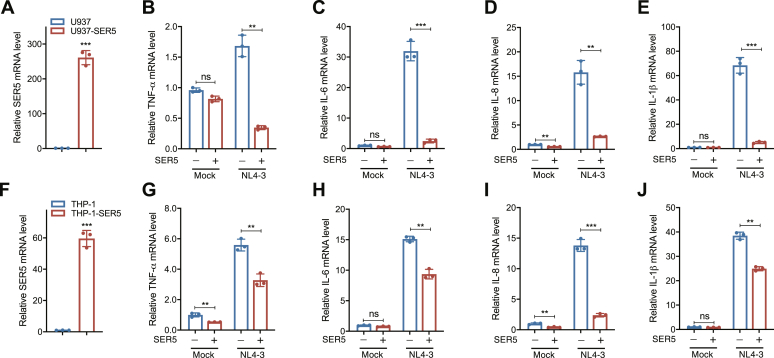
**SER5 downregulates the production of HIV-1 infection-induced anti-viral cytokines in U937 and THP-1 cells.***A* and *F*, The SER5 mRNA levels in U937 cells (*A*) and THP-1 cells (*F*) with or without SER5 stable expression. *B*–*E*, PMA-differentiated U937 cells and (*G*–*J*) THP-1 cells with or without SER5 stable expression were infected with VSV-G-pseudotyped NL4-3 for 48 h. The relative levels of mRNAs for TNF-α (*B*, *G*), IL-6 (*C*, *H*), IL-8 (*D*, *I*), and IL-1β (*E*, *J*) were then determined by RT-qPCR. In *A*–*J*, data are calculated from three independent experiments. ∗∗, *p* < 0.01; ∗∗∗, *p* < 0.001; ns, not significant (unpaired Student’s *t* test).

To further validate the inhibition effect of SER5 on the NF-κB signaling pathway during HIV-1 infection, U937 and THP-1 monocytic cells which are susceptible to HIV-1 were stably transduced with SER5 (U937-SER5 and THP-1-SER5) by lentivirus infection and the mRNA levels of SER5 were determined by RT-qPCR (Fig. 3, *A* and *F*). Subsequently, these cells were differentiated with PMA for 20 h followed by infection with VSV-G-pseudotyped NL4-3 for 48 h. We observed a significant reduction in the relative mRNA levels of TNF-α, interleukin (IL)-6, IL-8, and IL-1β, which are the downstream pro-inflammatory cytokines regulated by NF-κB signaling, upon stable expression of SER5 in U937 and THP-1 cells after the viral infection (Fig. 3, *B*–*E*, *G*–*J*), indicating that SER5 negatively regulates HIV-1-induced activation of NF-κB signaling in U937 and THP-1 cells.

### SER5 inhibits NF-κB-mediated HIV-1 transcription by blocking p65 recruitment to the LTR promoter

**Figure 4:**
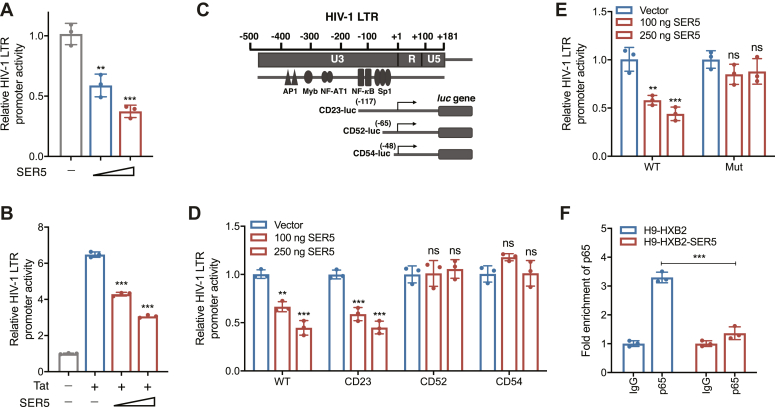
**SER5 inhibits NF-κB-mediated HIV-1 transcriptional activation.***A* and *B*, 100 ng or 250 ng of SER5 or pVR1012 were co-transfected with 300 ng pLTR-luciferase, 50 ng pRenilla-luciferase (*A*) and 200 ng of a Tat expression plasmid (*B*) into HEK293T cells for 36 h. The cells were harvested for assessment of the reporter gene expression by dual luciferase reporter assay. *C*, schematic representation of HIV-1 LTR and the truncated LTR-luciferase constructs. *D* and *E*, HEK293T cells were co-transfected with truncated (*D*) or mutated (*E*) pLTR-luciferase constructs, pRenilla-luciferase, and increasing doses of SER5 expression vector or pVR1012. The dual luciferase reporter assay was performed at 36 h post-transfection. *F*, the fold change enrichment of p65 by anti-p65 antibody or IgG was quantified at the HIV-1 LTR promoter in H9-HXB2 cells with or without stable SER5 expression using qPCR. In *A*, *B*, and *D*–*F*, data are calculated from three independent experiments. ∗, *p* < 0.05; ∗∗, *p* < 0.01; ∗∗∗, *p* < 0.001; ns, not significant (unpaired Student’s *t* test).

Activation of NF-κB signaling enhances the transcriptional activity of HIV-1 LTR due to the presence of an NF-κB-responsive element in its transcriptional control region, which can bind with the p50/p65 heterodimer (37, 38, 39, 48). Therefore, we investigated the impact of SER5 on the transcriptional activation driven by HIV-1 LTR using the dual luciferase reporter assay. The expression of LTR-driven luciferase was observed to decrease with increasing levels of SER5 (Fig. 4*A*), indicating an inhibitory effect of SER5 on HIV-1 LTR-driven transcriptional activation. Additionally, we examined the influence of SER5 on LTR promoter activity in the presence of the viral protein Tat, as successful HIV-1 transcription relies on Tat-LTR interaction (47, 49, 50). Consistently, a dose-dependent inhibition of HIV-1 LTR-driven gene expression by SER5 was observed in the presence of Tat (Fig. 4*B*).

Various widely expressed cellular transcription factors (such as Sp1 and TFIID) and inducible transcription factors (including NF-κB, NFAT, and AP-1) have been reported to bind specific regions within the LTR sequence and play a role in regulating HIV-1 transcription (38, 39, 51). Therefore, to determine whether the transcription factor NF-κB is responsive to SER5 at the level of LTR regulation, different LTR promoter-truncated constructs (52) were employed for the dual luciferase reporter assay in HEK293T cells as depicted schematically in Figure 4*C*. CD23-luc lacks the upstream regulatory regions of the LTR promoter but retains the enhancer elements (two NF-κB binding sites) and core regions (three SP1 binding sites) within its structure. CD52-luc only contains two SP1 binding sites from the core regions, while CD54-luc lacks all SP1 binding sites and includes only a small segment (−48 to +80) from the core regions. A dose-dependent inhibition of the promoter activity by SER5 were observed between the wild-type (WT) full-length LTR construct and the truncated mutant CD23-luc (Fig. 4*D*), indicating that the upstream region (5′-end to −117) of LTR does not contribute to SER5-mediated inhibition. In contrast, no significant reduction in the reporter activity was observed with SER5 in the CD52-luc and CD54-luc groups, suggesting that the site regulated by SER5 is located within the region containing the two NF-κB binding sites and one of the three SP1 binding sites. To further determine whether the SER5-mediated inhibition of HIV-1 transcription is NF-κB-dependent, we generated a luciferase reporter plasmid with mutations in both NF-κB binding sites within the LTR promoter (53, 54). As expected, while WT LTR activity was dose-dependently inhibited by SER5, this inhibitory effect was lost in the LTR NF-κB mutant (Fig. 4*E*).

We next investigated the impact of SER5 on the binding of p65 to LTR by Cut&tag analysis. The H9-HXB2 and H9-HXB2-SER5 cells were harvested and incubated with concanavalin A-coated magnetic beads, followed by incubation with anti-p65 or IgG primary antibody (Ab) and the secondary Ab. Then, Protein A/G fused with transposons was added for cutting the genome DNA near the target region, and the DNAs were extracted and subjected to qPCR. qPCR results using primers spanning from NF-κB to SP1 (−143 to +14)-responsive elements in the enhancer region revealed reduced recruitment of p65 to this region in H9-HXB2 cells stably expressing SER5 (Fig. 4*F*), compared to the significant enrichment observed when anti-p65 antibody was used over IgG in the control groups. These results indicate that SER5 inhibits HIV-1 transcription by blocking p65 recruitment to the NF-κB enhancer region in the LTR promoter.

### SER5 downregulates NF-κB signaling by promoting the degradation of MDA5 and RIG-I

**Figure 5:**
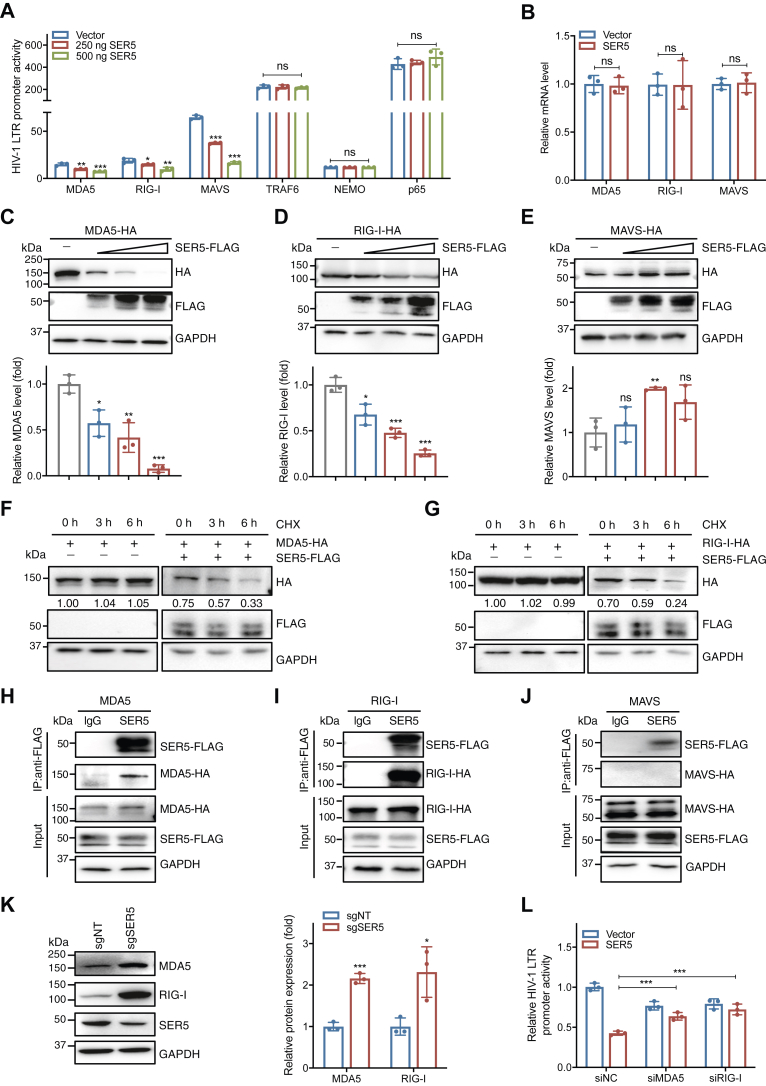
**SER5 downregulates NF-κB signaling by promoting the degradation of MDA5 and RIG-I.***A*, different doses of SER5 or pVR1012 were co-transfected with 300 ng pLTR-luciferase, 50 ng pRenilla-luciferase, and 200 ng of the expression plasmid of MDA5, RIG-I, MAVS, TRAF6, NEMO, or p65 into HEK293T cells. Cells were harvested 36 h later and assessment of the reporter gene expression by dual luciferase reporter assay. *B*, HEK293T cells transfected with 500 ng SER5 or pVR1012 were harvested 48 h post-transfection for detection of the endogenous mRNA levels of MDA5, RIG-I, and MAVS by RT-qPCR. *C*–*E*, HEK293T cells were co-transfected with 250 ng, 500 ng, or 1000 ng SER5 or pVR1012 and 500 ng MDA5 (*C*), RIG-1 (*D*), or MAVS (*E*). At 48 h post-transfection, the cell lysates were analyzed by Western blotting (*upper panel*). *Bottom panel*, the relative protein expression levels. (*F*, *G*) HEK293T cells were co-transfected with MDA5 (1 μg, F) or RIG-I (1 μg, *G*), along with SER5 (300 ng) or pVR1012 for 48 h. The cells were treated with CHX (50 μg/ml) for 3 h or 6 h before harvest and analyzed by Western blotting. *H*–*J*, HEK293T cells were co-transfected with 500 ng SER5 and 500 ng MDA5 (*H*), RIG-I (*I*), or MAVS (*J*) for 48 h. The co-immunoprecipitated proteins from the cell lysates were probed using anti-FLAG and anti-HA antibodies. (*K*) PBMCs activated with PHA for 72 h were infected with the sgSER5 or sgNT lentivirus and selected by puromycin, and the endogenous SER5, MDA5, and RIG-I expression were analyzed by Western blotting (*left panel*). *Right panel*, the relative protein expression levels. *L*, HEK293T cells were co-transfected with SER5 or pVR1012, pLTR-luciferase, and pRenilla-luciferase after transfection with the siRNAs targeting MDA5 or RIG-I for 24 h. The cells were harvested 24 h later for assessment of the reporter gene expression by dual luciferase reporter assay. In *C*–*G* and *K*, the indicated protein expression levels were calculated relative to the corresponding GAPDH levels. In (*A*–*E*), (*K*), and (*L*), data are calculated from three independent experiments. ∗, *p* < 0.05; ∗∗, *p* < 0.01; ∗∗∗, *p* < 0.001; ns, not significant (unpaired Student’s *t* test).

To dissect the steps at which SER5 interacted with the NF-κB signaling pathway, we co-transfected HEK293T cells with SER5 and the plasmid encoding one of the key molecules involved in this pathway. The results showed that SER5 significantly suppressed MDA5-, RIG-I-, and MAVS-induced HIV-1 LTR activation, while it had no effect on the downstream molecules TRAF6, NEMO, or p65-mediated enhancement (Fig. 5*A*). Further investigation revealed that SER5 did not influence the endogenous mRNA levels of MDA5, RIG-I, or MAVS (Fig. 5*B*), but dose-dependently reduced the protein expression levels of MDA5 and RIG-I (Fig. 5, *C*–*E*). The cycloheximide (CHX) chase assay showed that SER5 deficiency increased the half-life of MDA5 and RIG-I (Fig. 5, *F* and *G*), suggesting a role for SER5 in modulating the stability of these proteins. Co-immunoprecipitation analysis confirmed the interaction of SER5 with MDA5 and RIG-I (Fig. 5, *H* and *I*), whereas no interaction was observed with MAVS, TRAF6, NEMO, or p65 (Figs. 5*J*, S3, *A*–*C*). Conversely, knockout of SER5 expression in PBMCs resulted in elevated endogenous levels of MDA5 and RIG-I (Fig. 5*K*), suggesting that MDA5 and RIG-I play key roles as mediators in the downregulation activity of SER5 on the NF-κB signaling pathway. Furthermore, to validate that the downregulation activity exerted by SER5 on HIV-1 LTR-driven transcriptional activation depends on targeting MDA5 and RIG-I, their endogenous expression was silenced using siRNA transfection in HEK293T cells followed by Western blotting verification for the silencing efficiency (Fig. S3, *D* and *E*). The results confirmed that the absence of MDA5 or RIG-I significantly restored the SER5-mediated inhibition of LTR-dependent luciferase expression (Fig. 5*L*).

### SER5 mediates proteasomal degradation of MDA5 and RIG-I by recruiting TRIM40 to promote their K48-linked polyubiquitination

**Figure 6:**
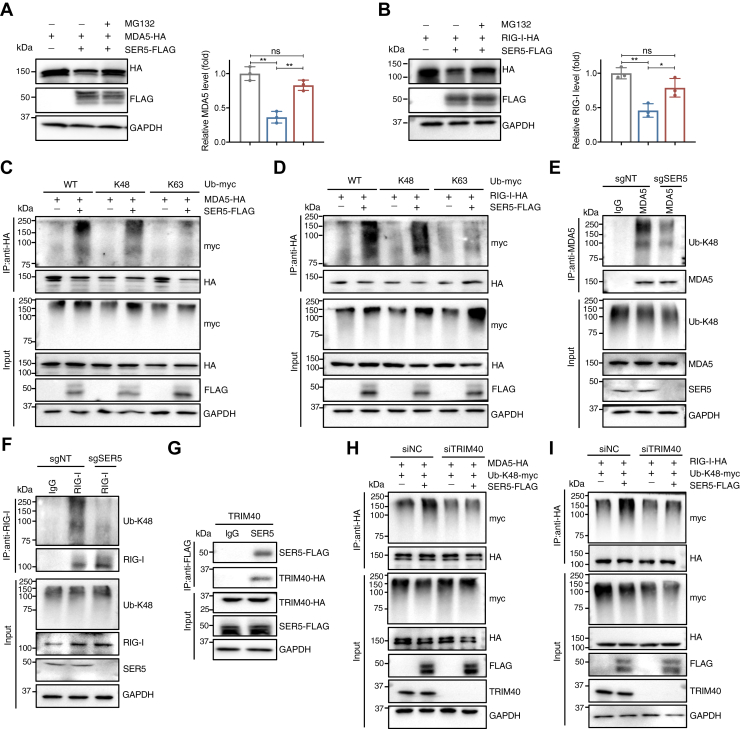
**SER5 mediates K48-linked polyubiquitination and proteasomal degradation of MDA5 and RIG-I.***A* and *B*, HEK293T cells were co-transfected with SER5 and MDA5 (*A*) or RIG-I (*B*) for 48 h. The cells were treated with 50 μM MG132 for 4 h before harvest and then analyzed by Western blotting (*left panel*). *Right panel*, the relative protein expression levels from three independent experiments were calculated relative to the corresponding GAPDH levels. ∗, *p* < 0.05; ∗∗, *p* < 0.01; ns, not significant (unpaired Student’s *t* test). *C* and *D*, plasmids encoding SER5, MDA5 or RIG-I, and WT Ub or lysine-mutated Ub that only retains the K48 or K63 residue were co-transfected into HEK293T cells. The ubiquitination of MDA5 (*C*) and RIG-I (*D*) was monitored by immunoprecipitation at 48 h post-transfection with 50 μM MG132 treatment for 4 h. *E* and *F*, SER5-knockout or control J-Lat 6.3 cells were treated with 50 μM MG132 for 4 h. The K48-linked ubiquitination of endogenous MDA5 (*E*) and RIG-I (*F*) was monitored by immunoprecipitation with a specific antibody against K48-linked Ub. *G*, HEK293T cells were co-transfected with 500 ng SER5 and 500 ng TRIM40 for 48 h. The cell lysates were immunoprecipitated with an anti-FLAG mAb and analyzed by Western blotting with anti-HA and anti-FLAG mAbs. *H* and *I*, HEK293T cells were co-transfected with siNC or siTRIM40 for 24 h. The cells were then co-transfected with SER5, Ub-K48, and MDA5 (*H*) or RIG-I (*I*), respectively. The ubiquitination of MDA5 and RIG-I was monitored by immunoprecipitation at 48 h post-transfection with 50 μM MG132 treatment for 4 h.

Next, to investigate the mechanism by which SER5 downregulates MDA5 and RIG-I protein levels, we co-transfected HEK293T cells with SER5 and either MDA5 or RIG-I, followed by treatment with a proteasomal inhibitor MG132 or a lysosomal inhibitor leupeptin. The reduction in MDA5 and RIG-I levels induced by SER5 was restored upon MG132 treatment (Fig. 6, *A* and *B*) but remained unaffected upon leupeptin treatment (Fig. S4, *A* and *B*), indicating that SER5 mediates the degradation of MDA5 and RIG-I through the proteasomal pathway.

Considering that ubiquitination is a key signal for proteasomal degradation, particularly *via* K48- and K63-linked polyubiquitin chains (55), we investigated whether the degradation of MDA5 and RIG-I mediated by SER5 could be competitively inhibited using dominant negative ubiquitin (Ub) mutants harboring mutations K48R or K63R. The results demonstrated that Ub-K48R blocked the SER5-mediated degradation of both MDA5 and RIG-I (Fig. S4, *C* and *D*), indicating that the K48-linked polyubiquitin chains primarily target these proteins for degradation. We next carried out ubiquitination assays by co-transfection of HEK293T cells with plasmids encoding SER5, MDA5 or RIG-I, and WT Ub or lysine-mutated Ub that only retains the K48 or K63 residue. The results showed that SER5 overexpression markedly increased K48-, but not K63-, linked polyubiquitination of RIG-I and MDA5 (Fig. 6, *C* and *D*). Conversely, knockdown of SER5 in J-Lat 6.3 cells reduced K48-linked polyubiquitination of endogenous MDA5 and RIG-I (Fig. 6, *E* and *F*). Collectively, these results suggest that SER5 promotes proteasomal degradation of MDA5 and RIG-I by facilitating their specific targeting through K48-linked polyubiquitination.

It has been reported that ring finger protein 125 (RNF125) or tripartite interaction motif 40 (TRIM40) attenuates the activation of RLRs by mediating K48-linked ubiquitination of MDA5 and RIG-I (56). Therefore, we investigated whether RNF125 or TRIM40 is the E3 ligase employed by SER5 to promote the degradation of MDA5 and RIG-I. Co-immunoprecipitation (co-IP) assays showed that SER5 interacts with TRIM40 (Fig. 6*G*) but not with RNF125 (Fig. S4*E*). To confirm whether TRIM40 plays a role in SER5-mediated polyubiquitination of MDA5 and RIG-I, we conducted ubiquitination assays in TRIM40-knockdown HEK293T cells. The knockdown of TRIM40 resulted in a decrease of K48-linked polyubiquitination of MDA5 and RIG-I induced by SER5, respectively (Fig. 6, *H* and *I*). These results suggested that SER5 recruits TRIM40 for the Ub K48-linked proteasomal degradation of MDA5 and RIG-I.

## Discussion

Current research has revealed that the majority of host restriction factors exert their antiviral functions through multiple pathways (57). While SER5 has been extensively reported as a viral entry inhibitor by incorporating into budding HIV-1 particles, recent studies have elucidated its role in blocking HIV-1 gene expression and influencing viral RNA capping processes (23, 24). These findings indicate that SER5 employs diverse antiviral mechanisms to restrict HIV-1 replication in host cells.

Here, we reported a novel function of SER5 in downregulating NF-κB signaling activation during HIV-1 infection, directly leading to the suppression of viral transcription by preventing the recruitment of the p50/p65 dimer to HIV-1 LTR. Consistent with the study of Shi *et al.* (24), our results demonstrate that SER5 reduces the expression of the Gag precursor protein Pr55 and capsid p24 in cells and virions through inhibition of viral transcription, consequently reducing viral production. This effect is observed across different coreceptor tropisms and subtypes of HIV-1 strains (Fig. S1*C*) as well as SIV (24). Notably, even in the absence of HIV-1 infection, SER5 significantly inhibits NF-κB activity induced by the key molecules involved in this signaling pathway in a dose-dependent manner (Figs. 2*E* and 5*A*) and markedly reduces the expression levels of several cytokines (Fig. 3). These results suggest that beyond its influence on viral infection-triggered NF-κB activation, SER5 also plays a physiological role in inhibiting NF-κB signaling activation independently. Therefore, the viral transcription inhibition function exhibited by SER5 during HIV-1 infection arises from its intrinsic ability to suppress NF-κB signaling.

Before these findings, the impact of SER5 on the NF-κB signaling pathway has not been consistently elucidated. One study demonstrated that SER5 enhances the IFN-I and NF-κB signaling pathways in human primary monocyte-derived macrophages upon SeV and poly(I: C) stimulation by interacting with MAVS and TRAF6 (25). Additionally, Pierini *et al.* reported that incorporation of SER5 into HIV-1 virions triggers proinflammatory cytokine production in myeloid target cells (58). However, a recent investigation utilizing THP-1 cell-differentiated macrophages revealed that while SER5 induces an elevation in IFN-I response, it does not activate the NF-κB response (23). Another study found that SER5 interacts with MDA5 but not with MAVS in PK15 and 3D4/2 cells (10). Our experiments also yielded negative results regarding the interaction between SER5 and MAVS or TRAF6 (Figs. 5*J*, S3*A*), raising questions about the choice of stimuli for activating NF-κB signaling. Although viral infection and various stimuli can activate NF-κB signaling through different mechanisms and induce gene expression related to inflammation and immunity, it is important to note that NF-κB-dependent transcription is not only tightly controlled by positive and negative regulatory mechanisms but also closely coordinated with the IFN-I signaling pathway (59). Therefore, previous observations of enhanced cytokine production mediated by SER5 may partly be an indirect consequence of either SER5-or stimuli-induced activation of IFN-I signaling. Our RNA-seq results provide support for the activation of IFN-I signaling by SER5 overexpression (Fig. S2*B*). Additionally, we propose that SER5 may regulate other signaling pathways involved in the replication process of HIV-1, such as the MAPK, PI3K-AKT, and JAK-STAT pathways (Figs. 2*A*, S2*C*), which could potentially lead to inconsistent outcomes under certain experimental conditions. In line with our findings, Zeng *et al.* also observed a decrease in TNF-α expression in the SER5-overexpression group when THP-1 cells were not activated by SeV infection (25). This consistency further supports our conclusion that SER5 may have a dynamic balancing function in promoting innate immune responses while downregulating NF-κB signaling.

However, one limitation of this study is that we could not fully mimic the physiological role of SER5 through *in vitro* experiments alone. The actual infection process of HIV-1 involves specific molecular interactions and feedback regulatory mechanisms (60). To address this limitation, we conducted repeated examinations on the activity of SER5 during viral infection and in HIV-1 susceptive or latently infected T lymphocytes, monocyte-derived macrophages, and PBMCs. We found that SER5 reduced nuclear accumulation of p65, suppressed p65 binding to the LTR and activation of HIV-1 transcription, and influenced the downstream pro-inflammatory cytokines regulated by NF-κB signaling. These findings strongly indicate an inhibitory effect exerted by SER5 on the NF-κB signaling pathway during HIV-1 infection.

**Figure 7:**
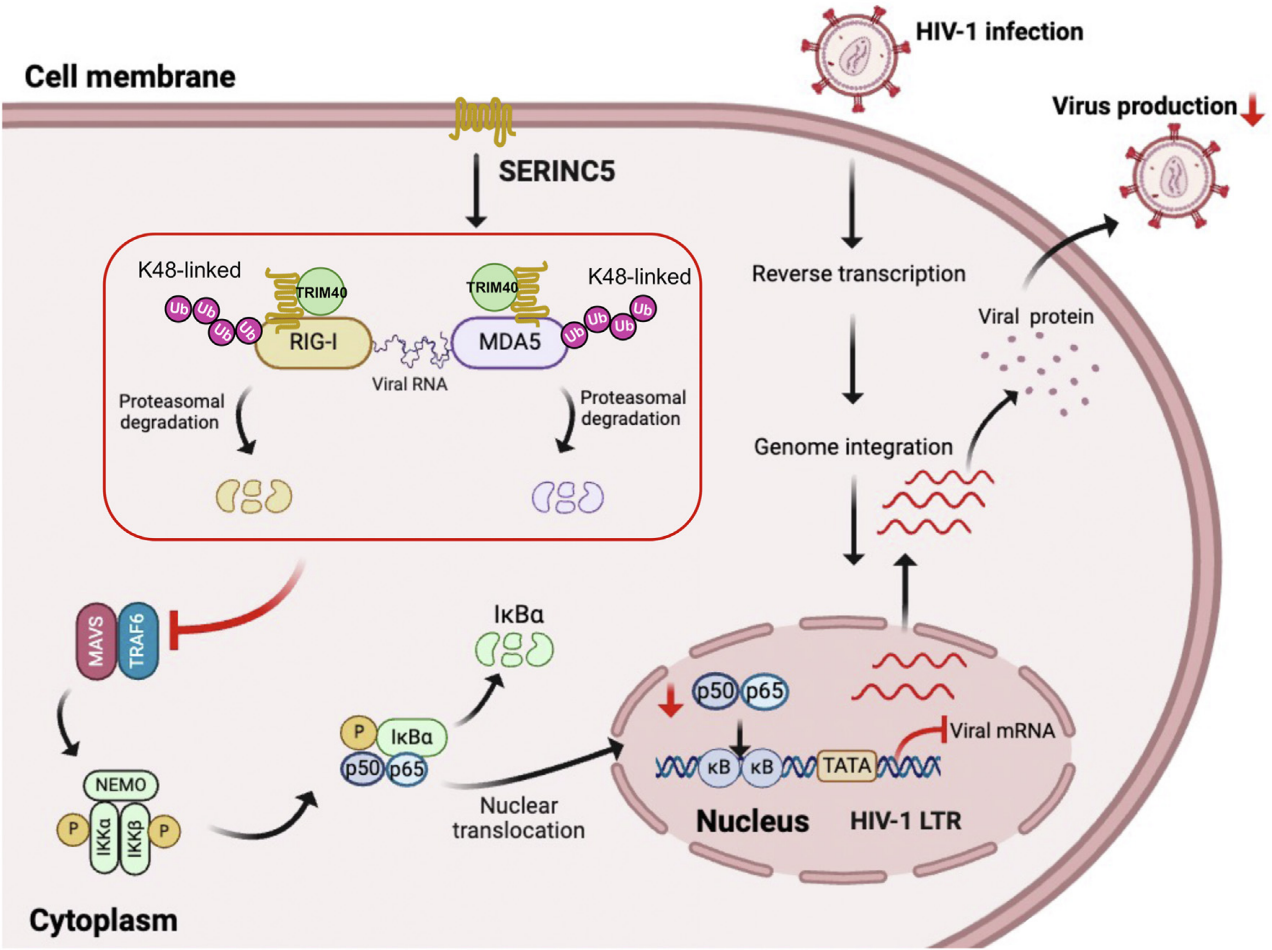
**Proposed mechanistic model showing restriction of HIV-1 replication by SER5.** SER5 inhibits NF-κB signaling by promoting the proteasomal degradation of MDA5 and RIG-I, thereby reducing the binding of the p50/p65 dimer to HIV-1 LTR in the nucleus, resulting in reduced viral production and increased genome latency. Mechanistically, SER5 interacts with MDA5 and RIG-I, recruiting TRIM40 as the E3 ubiquitination ligase to promote their K48-linked polyubiquitination and degradation.

Based on the previous and our findings, the antiviral landscape of SER5 can be inferred at different stages of HIV-1 replication. During the early stage following viral entry, SER5 activates innate immunity by positively regulating the IFN-I signaling pathway through IRF3 and IRF7 (25). This leads to the induction of the expression of hundreds of antiviral genes, enhancing host resistance against the virus. However, it also suppresses NF-κB signaling activation by promoting MDA5 and RIG-I degradation. After viral genome integration, SER5 inhibits the translocation of the p50/p65 dimer into the nucleus, thereby blocking its binding to HIV-1 LTR, resulting in reduced viral transcription and increased genome latency (Fig. 7). Then, the reduced viral production along with incorporation of SER5 in progeny virions collectively contribute to diminished intercellular viral spreading. Many host antiviral restriction factors are produced in response to IFNs or encoded by IFN-stimulated genes, such as APOBEC3, Tetherin, TRIM5α, and SAMHD1 (61), which provide regulatory positive or negative feedback on innate immunity. However, the production of SER5 is not IFN, pro-inflammatory ILs, or TNF-α inducible and is upregulated during myeloid cell differentiation (62, 63). Here, we demonstrate that the expression of SER5 can be triggered by HIV-1 infection and fluctuates with viral latency status (Fig. 1*I*). This suggests that SER5 may be involved in the transcriptional regulation mechanisms of host genes and its role in regulating the innate immune pathways extends beyond a mere response to viral infection.

We demonstrated the interactions of SER5 with both MDA5 and RIG-I, but not with the other downstream molecules in the NF-κB signaling pathway. SER5 can localize to various membrane structures, including the nuclear membrane and endoplasmic reticulum (64, 65), suggesting that it may encounter MDA5 and RIG-I in the cytoplasm during transportation to the cell membrane. Considering that MDA5 and RIG-I are also involved in mediating IFN-I signaling, degradation by SER5 would impact their role in activating IFN-I responses. This suggests that the positive effect of SER5 on the IFN-I signaling pathway is not solely dependent on these RLRs and may extend beyond its role in host defense against RNA viruses. Mechanistically, we found that SER5 recruits TRIM40 as the E3 ubiquitination ligase, to promote K48-linked polyubiquitination and degradation of MDA5 and RIG-I through proteasomal pathways (Fig. 6).

Collectively, our findings strongly suggest that SER5 exerts a suppressive effect on HIV-1 replication by inhibiting NF-κB-mediated viral gene expression. Therefore, SER5 serves as an important host defense factor with diverse antiviral mechanisms in HIV-1 target cells and a coordinator of innate immunity. The findings in this study offer valuable insights for the development of novel therapeutic strategies against HIV-1.

## Materials and methods

### Human participants

Blood was obtained from three healthy donors under the clinical protocols approved by the Institutional Review Boards of the First Hospital of Jilin University (No: 20K097–002). The experiments conformed to the principles set out in the WMA Declaration of Helsinki and the Department of Health and Human Services Belmont Report. The participants including one female and two male subjects provided written informed consents. PBMCs were isolated from the blood by the Lymphoprep density gradient centrifugation (STEMCELL Technologies, Beijing, China).

### Cell lines

HEK293T cells (CRL-11268) were purchased from the American Type Culture Collection (ATCC, Manassas, VA). HeLa-derived indicator TZM-bl cells, THP-1 cells, U937 cells, and J-Lat 6.3 cells were provided by the National Institutes of Health, HIV Reagent Program (NIH-HRP). The chronically infected cell line H9-HXB2 was described previously (66, 67). HEK293T and TZM-bl cells were maintained in Dulbecco’s modified Eagle’s medium (DMEM) supplemented with 10% fetal bovine serum (FBS) at 37 °C in 5% CO_2_. H9-HXB2, THP-1, U937, and J-Lat 6.3 cells were maintained in Roswell Park Memorial Institute (RPMI) 1640 medium with 10% FBS at 37 °C and 5% CO_2_. THP-1 and U937 cells were differentiated with phorbol 12-myristate 13-acetate PMA (50 ng/ml) for 20 h before use. PBMCs were activated with 5 μg/ml phytohemagglutinin (PHA) (Roche, Merck) for 72 h and maintained in RPMI 1640 medium with 20 units/ml human IL-2 (Roche) at 37 °C and 5% CO_2_.

### Plasmids

The infectious molecular clone pNL4-3, pNL4-3-Luc.R^−^E^−^, and pHXB2, as well as pVR1012-BCA2 and pNF-κB-luciferase, have been previously described (68, 69, 70, 71). The pLTR-luciferase and pRenilla-luciferase reporter plasmids were kindly provided by Dr. Wenyan Zhang (72). The different proviral expression plasmids of the HIV-1 clones were obtained from NIH-HRP.

SER5 (no. NM_032861.43) fragments were amplified through PCR with cDNA from HepG2 cells serving as the template. Then the fragments were inserted into pVR1012 or pLVX vectors. The truncated mutants of HIV-1 LTR were generated by PCR using pLTR-luciferase as the template. DNA sequence coding for p65 (UniProtKB accession no. P98152) was synthesized (GenScript, Piscataway, NJ) and inserted into the pVR1012 vector with a V5 tag. The MDA5, RIG-I, MAVS, TRIM40, and RNF125 fragments were amplified from pcDNA3.1-MDA5-HA, pCMV-RIG-I-HA, pcDNA3.1-MAVS-HA, pCMV-TRIM40-HA, and pCMV-RNF125-HA, respectively, which were purchased from MiaoLingBio (Wuhan, China), and inserted into the pVR1012 vector with an HA tag. The NEMO and TRAF6 fragments were amplified from pDONR223-NEMO and pCDNA-TRAF6, respectively, which were purchased from Addgene, and inserted into the pVR1012 vector with an HA tag. Ub-myc, Ub-K48R-myc, Ub-K63R-myc, Ub-K48-myc, and Ub-K63-myc fragments were synthesized (GenScript) and inserted into the pVR1012 vector.

### Antibodies, chemicals, siRNAs, and primers

Antibodies are listed in Table S1 and were all validated for specificity prior to use by comparison with mock cell samples; Chemicals are listed in Table S2; siRNAs and primers in Table S3.

### Lentiviral production and generation of SER5-stably expressing cell lines

DNA transfection was carried out using a jetPRIME transfection reagent (Polyplus transfection) according to the manufacturer’s instructions. Lenti-SER5 pseudoviruses were produced by transfection of HEK293T cells with pLVX-SER5, the backbone plasmid psPAX2 (Addgene), and pVSV-G for 48 h. Lentiviruses were harvested and quantified using an HIV-1 p24 ELISA kit (Xpressbio, Frederick, MD) to infect H9-HXB2, THP-1, or U937 cells. At 24 h post-infection, puromycin (0.4 μg/ml) was added to the medium for selection. To remove the dead cells, the cell culture medium was replaced every 2 days. Seven to 10 days later, the expression of SER5 was detected by RT-qPCR.

### Generation of knockout cell lines and small-interfering RNA interference

To generate SER5-knockout J-Lat 6.3 cells or PBMCs, sgRNA oligos were cloned into a LentiCRISPR V2 shuttle vector (Addgene). The shuttle plasmid carrying sgRNA was co-transfected with psPAX2 and pVSV-G into HEK293T cells to produce pseudoviruses targeting SER5 (sgSER5) or a random negative target (sgNT). J-Lat 6.3 cells were infected with 1 ng p24 of sgSER5 or sgNT pseudoviruses for 24 h with 20 μg/ml DEAE-dextran (Sigma-Aldrich, Merck). Then, the medium was changed to RPMI 1640 containing 0.4 μg/ml puromycin for selection of 7 days. PHA-activated PBMCs were infected with 1 ng p24 of sgSER5 or sgNT pseudoviruses for 48 h with 20 μg/ml DEAE-dextran, followed by selection with puromycin (0.5 μg/ml) for 3 days. To knock down SER5, MDA5, RIG-I, or TRIM40 expression, HEK293T cells were transfected with the small interfering RNA (siRNA) targeting SER5, MDA5, RIG-I, TRIM40, or a random negative control gene (siNC) according to the manufacturer’s instructions (GenePharma).

### HIV-1 production, purification, and infection

HIV-1 virus was produced by transfecting pNL4-3 into HEK293T cells in 6-well plates. VSV-G-pseudotyped NL4-3 was produced by co-transfecting pNL4-3-Luc.R^−^E^−^ and pVSV-G into HEK293T cells. Forty-eight hours later, the supernatants were collected and precleared of cellular debris by centrifugation at 1000*g* for 10 min at 4 °C. Virus particles were then concentrated through a 20% sucrose cushion by ultracentrifugation at 100,000*g* for 2 h. The viral pellets were resuspended in 15 μl RIPA lysis buffer (pH 7.4, containing 50 mmol/L Tris-HCl, 150 mmol/L NaCl, 1% Triton X-100, 1% sodium deoxycholate, 0.1% SDS, and 1 mmol/L EDTA) for immunoblotting or in phosphate-buffered saline (PBS) for virus titration by viral p24 ELISA. TZM-bl indicator cells containing an integrated HIV-1 LTR promoter-luciferase gene were used to assess infectious HIV-1 production. Viruses were mixed with DEAE-dextran at a final concentration of 20 μg/ml and then incubated with 1 × 10^4^ TZM-bl cells per well in 96-well plates. Infectivity was measured at 48 h after infection by a luciferase assay according to the manufacturer’s instructions (Promega). For infection of THP-1 and U937 cells or PBMCs, 5 × 10^5^ or 1 × 10^6^ cells were incubated with VSV-G-pseudotyped NL4-3 of 1 ng p24.

### Western blotting

Cells were harvested at 48 h after transfection, centrifuged at 1000*g* for 5 min to remove the supernatant, and lysed with 30 μl cytobuster (Merck) on ice for 30 min, followed by centrifugation at 13,600*g* for 10 min at room temperature. Then, the supernatant was collected and mixed with the loading buffer. The prepared protein samples were separated by SDS-polyacrylamide gel electrophoresis (SDS-PAGE), followed by transferred onto nitrocellulose-membranes. After blocking in 5% nonfat milk, the membranes were probed with primary and secondary antibodies, incubated with an ECL luminescence reagent (Tiangen) for 1 min, and then exposed using a Tanon 5200 Chemiluminescence Imaging System (Tanon). Protein band intensities were determined by Adobe Photoshop CC 2019 software.

### RNA extraction, RT-qPCR, and RNA-seq analysis

RNA was isolated with a TRIzol reagent (TransGen Biotech). cDNA was synthesized from 250 to 1000 ng of total RNA using a TransScript All-in-One First-Strand cDNA Synthesis SuperMix kit (TransGen Biotech). The SYBR green-based RT-qPCR was conducted by a TransStart Top Green qPCR SuperMix kit (TransGen Biotech). All RT-qPCR experiments were performed in triplicates. The mRNA levels were normalized to GAPDH mRNA to determine the relative expression ratio.

For RNA-seq analysis, SER5 was overexpressed or knocked down in HEK293T cells in the presence of pNL4-3. The harvested cells were mixed with TRIzol and then sent to Novogene Bioinformatics Technology for sequencing. For pathway activity analysis, gene expression data from each sample were implemented in an R “GSVA” package (https://github.com/rcastelo/GSVA) and “clusterProfiler” package (https://github.com/YuLab-SMU/clusterProfiler) to evaluate the enrichment of related gene sets. To identify mRNAs that were differentially expressed between the SER5 group and control group, an R “DESeq2” package (https://github.com/thelovelab/DESeq2) was used for batch adjusting.

### Proteome profiling

The proteome profiler antibody microarray analysis was performed with a Proteome Profiler Human NF-κB Pathway Array (R&D Systems) according to the manufacturer’s protocol. Briefly, each sample was incubated overnight at 4 °C on the dot blot membrane of the human NF-κB pathway array. The membrane was washed, incubated with a reconstituted detection antibody cocktail and HRP-conjugated streptavidin, and exposed using the Tanon 5200 Chemiluminescence Imaging System. The mean intensities of each spot were quantified using Image-J software (LOCI, University of Wisconsin).

### Dual-luciferase reporter assay

HEK293T cells were plated into 24-well plates and transfected at the following day with 300 ng of the reporter plasmid for NF-κB or HIV-1-LTR promoter, 50 ng of pRenilla-luciferase, and different interested protein expression plasmids per well. At 24 h after transfection, the transactivation activity of the promoters was determined by measuring Firefly and Renilla luciferase activities using a Dual-Luciferase Reporter assay kit (Promega). The relative promoter activity was calculated by normalizing the Firefly luciferase activity to the Renilla luciferase activity.

### Nuclear and cytoplasmic extracts

The method for nuclear and cytoplasmic extracts has been described previously (73). Briefly, cells were first resuspended in 80 μl of RLN buffer (pH 7.4, containing 50 mmol/L Tris-HCl, 40 mmol/L NaCl, 1.5 mmol/L MgCl_2_, and 0.5% Nonidet P-40) in the presence of protease inhibitor (Roche) and incubated on ice for 8 min to extract the supernatants as cytoplasmic samples. The nuclear pellets were washed once with the RLN buffer and then resuspended in 20 μl RIPA buffer. After vortexed for 30 min, the nuclear samples were centrifuged to remove debris. Then, the samples were separated by SDS-PAGE and detected by immunoblotting.

### Cut&tag assay

Cut&tag (Vazyme) is a method to extract the DNA bound to protein (74). The cells were harvested and incubated with 10 μl concanavalin A-coated magnetic beads at room temperature for 10 min, followed by addition of 3 μg anti-p65 or IgG primary Ab at 4 °C overnight. After the supernatant was removed, the secondary Ab was added to incubate with the cell (nucleus)-magnetic bead complex at room temperature for 30 to 60 min. Then, the supernatant was discarded, followed by addition of 100 μl Protein A/G fused with transposons for cutting the genome DNA near the target region. Then, the DNAs were extracted and subjected to qPCR. All qPCR DNA signals were normalized to the signals obtained from the IgG-enriched group.

### CHX chase, co-IP, and ubiquitination assays

For the CHX chase assay, transfected HEK293T cells were treated with CHX (50 μg/ml) for 3 h or 6 h before harvest and analyzed by Western blotting. For co-IP and ubiquitination assays, cells in 6-well plates were harvested at 48 h after transfection and washed twice with cold PBS, then lysed in 200 μl cytobuster supplemented with Complete protease inhibitor cocktail tablets (EDTA Free, Roche) at 4 °C for 60 min. The cell lysates were incubated with 3 μg target protein-specific primary Ab or IgG monoclonal Ab at 4 °C overnight and then incubated with 30 μl Protein G agarose beads (Roche) at 4 °C for 4 h. The beads were washed three times with washing buffer, boiled in SDS sample buffer for Western blotting. To assess the ubiquitination status of MDA5 and RIG-I, the transfected HEK293T cells or SER5-knockout or control J-Lat 6.3 cells were treated with 50 μM MG132 for 4 h before harvest.

### Statistical analysis

Data are shown as mean ± standard deviation (SD). Significance is analyzed by using unpaired Student’s *t* test or repeated-measured ANOVA with PRISM v6 (GraphPad Software, Inc.). In all figures, ∗ indicates *p* < 0.05; ∗∗, *p* < 0.01; ∗∗∗, *p* < 0.001; ns indicates no significance.

## Data availability

The RNA-seq data generated during the study is available at NCBI at https://www.ncbi.nlm.nih.gov/sra/PRJNA1072810 and NCBI Sequence Read Archive (SRA) ID PRJNA1072810.

The Data generated during the study is available at figshare at https://doi.org/10.6084/m9.figshare.28092131.v1.

## Supporting information

This article contains support information.

## Conflict of interest

The authors declare that they have no conflicts of interest with the contents of this article.
