# Supplementary material for: The host restriction factor SERINC5 inhibits HIV-1 transcription by negatively regulating NF-κB signaling
Source: J Biol Chem. 2024 Dec 7;301(1):108058. doi: 10.1016/j.jbc.2024.108058 (PMC11750542; doi:10.1016/j.jbc.2024.108058)
Supplement: Supplemental Material [file mmc1.pdf]

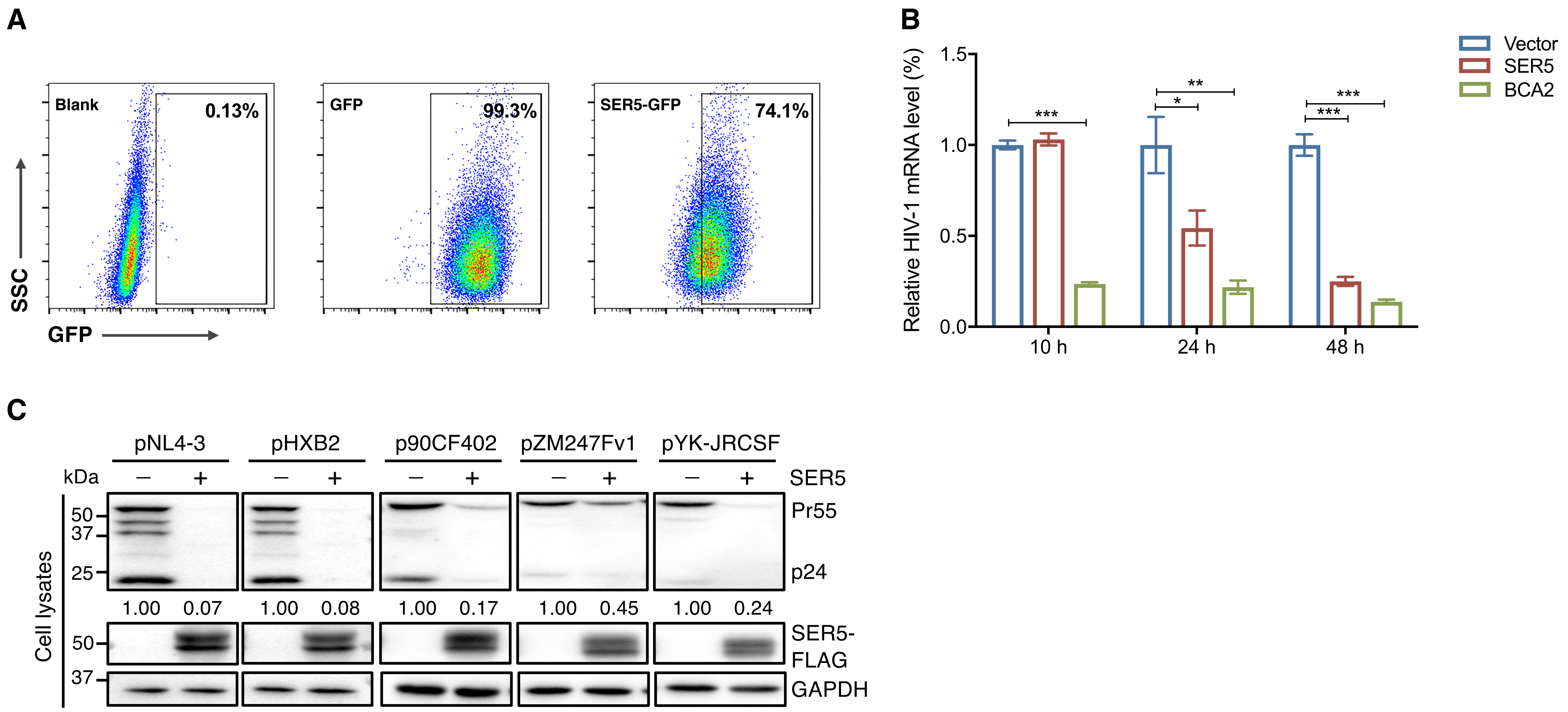

Supplementary Figure 1. The anti-viral gene expression ability of SER5.
(A) The percentage of GFP-positive H9-HXB2 cells infected by the Lenti-GFP or Lenti-SER5-GFP pseudovirus was detected by flow cytometry. **(B)** HEK293T cells were co-transfected with pNL4-3 and SER5, BCA2, or pVR1012. The levels of HIV-1 mRNA probed to *nef* were analyzed at 10, 24, and 48 h after transfection. **(C)** HEK293T cells were co-transfected with the proviral expression plasmids of different HIV-1 clones and SER5 or pVR1012. The cells were harvested at 48 h post-transfection and analyzed by Western blotting. The viral p24 levels were calculated relative to GAPDH levels. In B, *, $p < 0.05$; **, $p < 0.01$; ***, $p < 0.001$ (unpaired Student's t-test).

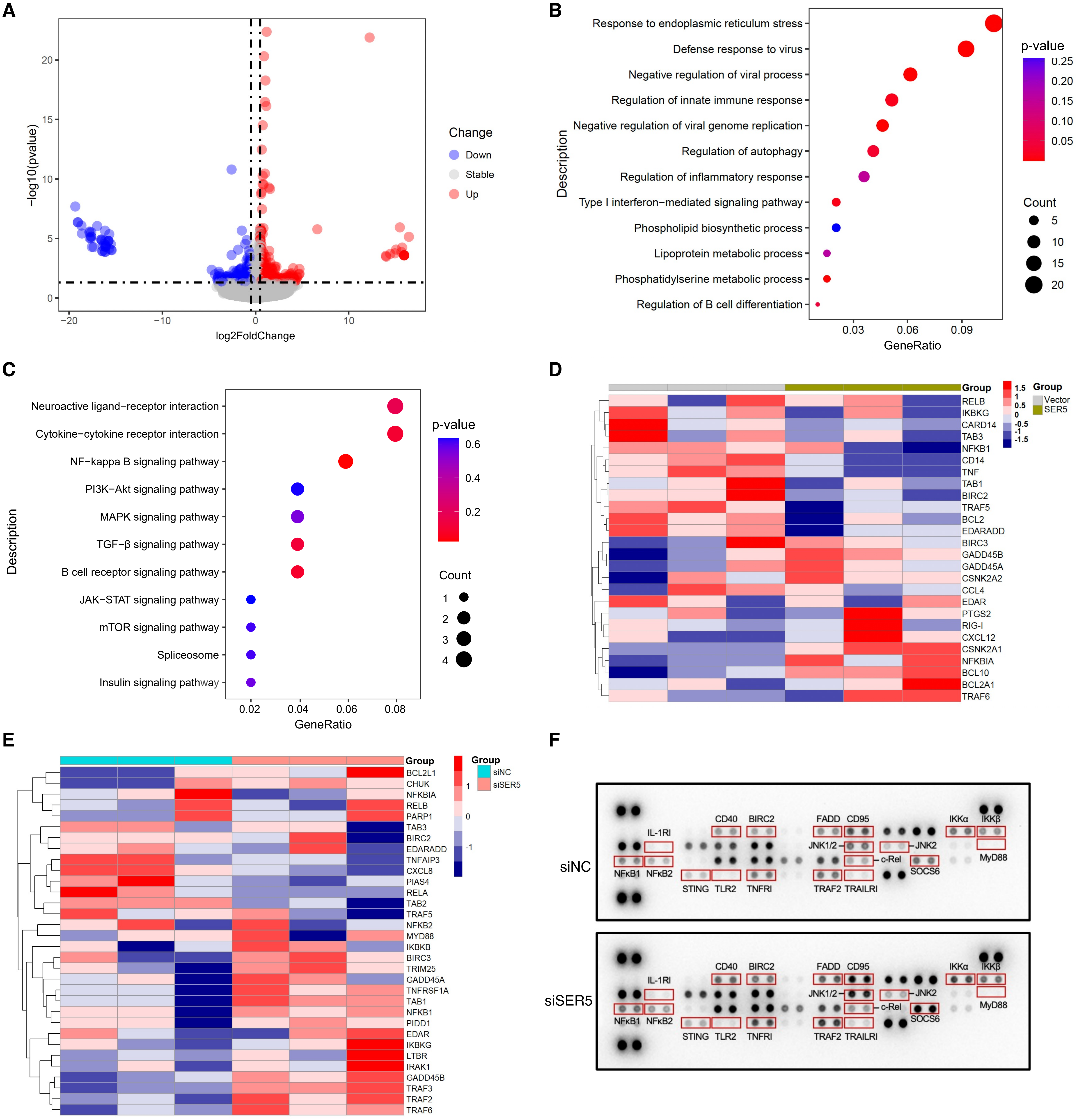

A
-log10(pvalue)
log2FoldChange
Change
Down
Stable
Up
B
Response to endoplasmic reticulum stress
Defense response to virus
Negative regulation of viral process
Regulation of innate immune response
Negative regulation of viral genome replication
Regulation of autophagy
Regulation of inflammatory response
Type I interferon−mediated signaling pathway
Phospholipid biosynthetic process
Lipoprotein metabolic process
Phosphatidylserine metabolic process
Regulation of B cell differentiation
Description
GeneRatio
p-value
Count
C
Neuroactive ligand−receptor interaction
Cytokine−cytokine receptor interaction
NF−kappa B signaling pathway
PI3K−Akt signaling pathway
MAPK signaling pathway
TGF−β signaling pathway
B cell receptor signaling pathway
JAK−STAT signaling pathway
mTOR signaling pathway
Spliceosome
Insulin signaling pathway
Description
GeneRatio
p-value
Count
D
Group
Vector
SER5
RELB
IKBKG
CARD14
TAB3
NFKB1
CD14
TNF
TAB1
BIRC2
TRAF5
BCL2
EDARADD
BIRC3
GADD45B
GADD45A
CSNK2A2
CCL4
EDAR
PTGS2
RIG-I
CXCL12
CSNK2A1
NFKBIA
BCL10
BCL2A1
TRAF6
E
Group
siNC
siSER5
BCL2L1
CHUK
NFKBIA
RELB
PARP1
TAB3
BIRC2
EDARADD
TNFAIP3
CXCL8
PIAS4
RELA
TAB2
TRAF5
NFKB2
MYD88
IKBKB
BIRC3
TRIM25
GADD45A
TNFRSF1A
TAB1
NFKB1
PIDD1
EDAR
IKBKG
LTBR
IRAK1
GADD45B
TRAF3
TRAF2
TRAF6
F
siNC
siSER5
IL-1RI
CD40
BIRC2
FADD
CD95
IKKα
IKKβ
JNK1/2
JNK2
c-Rel
MyD88
SOCS6
NFκB1
NFκB2
STING
TLR2
TNFRI
TRAF2
TRAILRI

Supplementary Figure 2. Profiling of the transcripts and the NF-κB signaling pathway-related proteins in SER5-overexpressed or knockdown HEK293T cells.

(A to C) RNA-seq analysis of the transcripts in SER5-overexpressed HEK293T cells in the presence of pNL4-3. The analysis was performed in triplicates by Volcano plots (A) and KEGG pathway enrichment analysis of upregulated (B) and downregulated (C) differentially-expressed genes (DEGs) from each comparison. Point size indicates the DEG number (the bigger dots refer to larger amounts). GeneRatio refers to the value of enrichment factor which is the quotient of the number of DEGs and total gene amount in that pathway. **(D)** Heatmap showing relative gene expression of the NF-κB signaling pathway-related genes involved in SER5 overexpression by GSVA. **(E, F)** Heatmap analysis of relative gene expression of the NF-κB signaling pathway-related genes (E) and proteins (F) in SER5-knockdown and control HEK293T cells pre-transfected with pNL4-3. Spots of proteins with increased expression are marked in red rectangles.

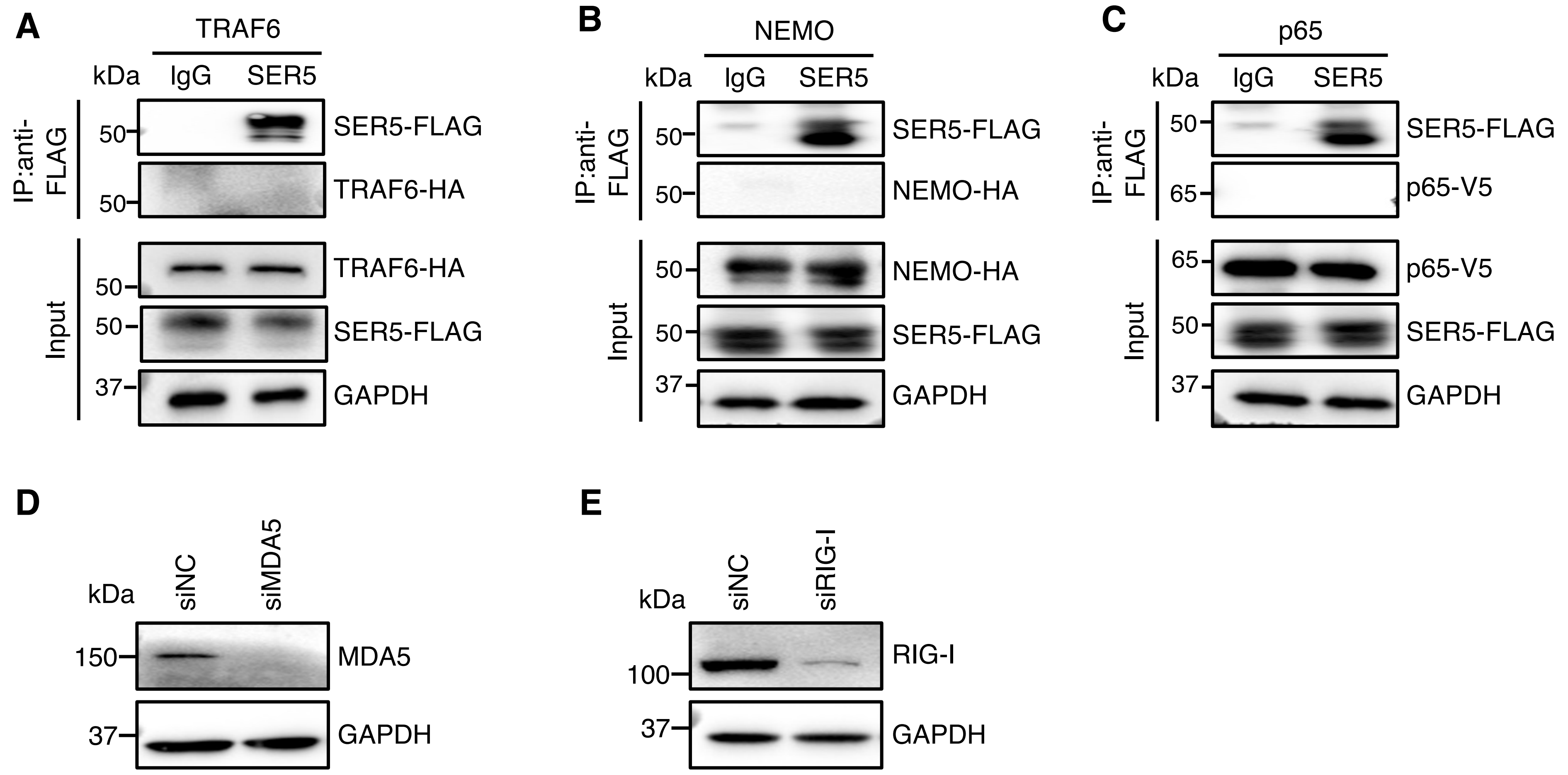

Supplementary Figure 3. The interaction between SER5 and TRAF6, NEMO, or p65.

(A to C) HEK293T cells were co-transfected with SER5 and TRAF6 (A), NEMO (B), or p65 (C), and co-immunoprecipitated proteins from the cell lysates were probed using anti-FLAG and anti-HA or anti-V5 mAbs. **(D, E)** The siRNA silencing efficiency of MDA5 (D) and RIG-I (E) in HEK293T cells was assessed by Western blotting.

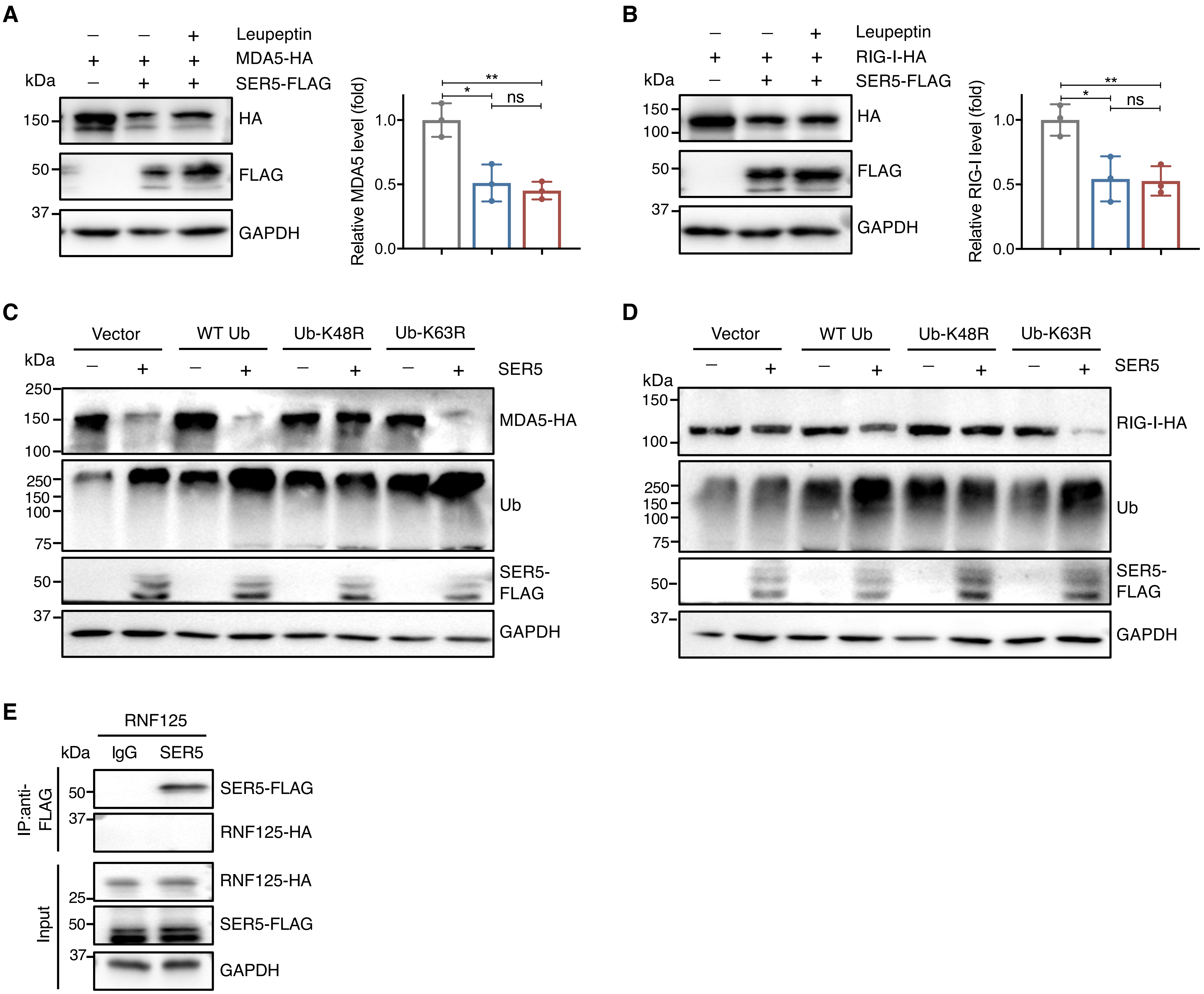

Supplementary Figure 4. K48-linked polyubiquitination of MDA5 and RIG-I mediated by SER5 was independent of lysosome and RNF125.

(A, B) HEK293T cells were co-transfected with SER5 and MDA5 (A) or RIG-I (B) for 48 h. The cells were treated with 10 μM leupeptin for 24 h before harvest and then analyzed by Western blotting (left panel). Right panel, the relative protein expression levels from three independent experiments were calculated relative to the corresponding GAPDH levels. **(C, D)** 500 ng SER5 or pVR1012 was co-transfected with 500 ng pVR1012, WT ubiquitin (Ub), Ub-K48R, or Ub-K63R and 500 ng MDA5 (C) or RIG-I (D) into HEK293T cells. The cells were analyzed by Western blotting at 48 h post-transfection with anti-HA and anti-FLAG mAbs and a specific antibody against Ub. **(E)** HEK293T cells were co-transfected with 500 ng SER5 and 500 ng RNF125 for 48 h. The cell lysates were immunoprecipitated with an anti-FLAG mAb and analyzed by Western blotting with anti-HA and anti-FLAG mAbs. In A and B, *, $p < 0.05$; **, $p < 0.01$; ns, not significant (unpaired Student's t-test).
