# Supplementary material for: The host restriction factor SERINC5 inhibits HIV-1 transcription by negatively regulating NF-κB signaling
Source: J Biol Chem. 2024 Dec 7;301(1):108058. doi: 10.1016/j.jbc.2024.108058 (PMC11750542; doi:10.1016/j.jbc.2024.108058)
Supplement: Supporting information [file mmc2.docx]

**Supporting Information Table**

**Table S1.** Information of antibodies. The detailed information of antibodies is provided, including sources, catalog numbers and dilutions.

| **Name** | **Source** | **Catalog Number** | **Dilution** |
| --- | --- | --- | --- |
| Anti-FLAG | Sigma-Aldrich | F1804 | WB: 1:10000  IP: 1:100 |
| Anti-HA | Covance | 901514 | WB: 1:10000  IP: 1:100 |
| Anti-GAPDH | Proteintech | 60004-1-Ig | WB: 1:10000 |
| Anti-IκBα | Proteintech | 10268-1-AP | WB: 1:1000 |
| Anti-p-IκBα | Affinity | AF2002 | WB: 1:500 |
| Anti-p65 | Proteintech | 66535-1-Ig | WB: 1:1000 |
| Anti-p-p65 | Santa Cruz | sc-136548 | WB: 1:200 |
| Anti-Histone H3 | Proteintech | 17168-1-AP | WB: 1:1000 |
| Anti-Ub | Santa Cruz Biotechnology | Sc-166553 | WB: 1:500 |
| Anti-Ub-K48 | Cell Signaling Technology | 4289 | WB: 1:1000 |
| Anti-p24 | NIH HIV reagent program | ARP-6521 | WB: 1:1000 |
| Anti-MDA5 | abcam | ab126630 | WB: 1:1000  IP: 1:100 |
| Anti-RIG-I | abcam | ab180675 | WB: 1:5000  IP: 1:100 |
| Rabbit polyclonal anti-SERINC5 | abcam | ab204400 | WB: 1:500 |
| Anti-TRIM40 | proteintech | 24526-1-AP | WB: 1:1000 |
| Normal mouse IgG | Santa Cruz Biotechnology | Sc-2025 | IP: 1:100 |
| Normal Rabbit IgG | Beyotime | A7016 | IP: 1:100 |
| Goat Anti-Mouse-HRP IgG | Jackson ImmunoResearch | 115-035-003 | WB: 1:10000 |
| Goat Anti-Rabbit-HRP IgG | Jackson ImmunoResearch | 111-005-003 | WB: 1:10000 |

**Table S2.** Information of chemicals. The detailed information of chemicals is provided, including sources and catalog numbers.

| **Name** | **Source** | **Catalog Number** |
| --- | --- | --- |
| Enfuvirtide | Yuanye Bio-Technology Co., Ltd | CRL-11268 |
| MG132 | Sigma-Aldrich | M8699 |
| Leupeptin | Sigma-Aldrich | L8511 |
| PHA | Roche | 11249738001 |
| IL-2 | Roche | 10799068001 |
| PMA | Beyotime | S1819 |
| CHX | MCE | HY-12320 |

**Table S3.** Primers and siRNA used in this study

| **Primer** | **Forward primer (5’-3’)** | **Experiment** |
| --- | --- | --- |
| SERINC5-F | TGGGATATT CTGCCGTGTATAGA | RT-qPCR |
| SERINC5-R | GGTGTCCTGATCTGGAATGAAG |  |
| Nef-F | GTACCAGTTGAGCCAGATAAGG | RT-qPCR |
| Nef-R | GCTGTCAAACCTCCACTCTAAC |  |
| GAPDH-F | TGGAGTCCACTGGTGTCTTCAC | RT-qPCR |
| GAPDH-R | TTCACGCCCATCACAAACA |  |
| MDA5-F | TCACAAGTTGATGGTCCTCAAGT | RT-qPCR |
| MDA5-R | CTGATGAGTTATTCTCCATGCCC |  |
| RIG-I-F | TGTGCTCCTACAGGTTGTGGA | RT-qPCR |
| RIG-I-R | CACTGGGATCTGATTCGCAAAA |  |
| MAVS-F | TTCTAATGCGCTCACCAATCC | RT-qPCR |
| MAVS-R | CCATGCTAGTAGGCACTTTGGA |  |
| EDAR-F | CAGCCCGAGCGGAATACTC | RT-qPCR |
| EDAR-R | CCGTAGCCACAGGACAGGTA |  |
| EDARADD-F | CCATTCAAGATACGGAACTCCC | RT-qPCR |
| EDARADD-R | AGCAAGTCACTTATGGTGGGG |  |
| TRAF2-F | CCTTCCCAGATAATGCTGCCC | RT-qPCR |
| TRAF2-R | GCTCTCGTATTCTTTCAGGGTC |  |
| TRAF3-F | CAGACTAACCCGCCGCTAAAG | RT-qPCR |
| TRAF3-R | GATGCTCTCTTGACACGCTGT |  |
| TRAF5-F | GACCTGAAAGAGCATTTGAGTGC | RT-qPCR |
| TRAF5-R | AGTGCTTAAAAGGACAGTCTTGC |  |
| TRAF6-F | TTTGCTCTTATGGATTGTCCCC | RT-qPCR |
| TRAF6-R | CATTGATGCAGCACAGTTGTC |  |
| TAB1-F | AACTGCTTCCTGTATGGGGTC | RT-qPCR |
| TAB1-R | AAGGCGTCGTCAATGGACTC |  |
| TAB3-F | AGCAGCCCACAGCTTGATATT | RT-qPCR |
| TAB3-R | ACTAGGAGAATGGATACCCAGGT |  |
| NEMO-F | AAGAGCCAACTGTGTGAGATG | RT-qPCR |
| NEMO-R | TTCGCCCAGTACGTCCTGA |  |
| IκBα-F | GCTATTCTCCCTACCAGCTC | RT-qPCR |
| IκBα-R | CAGTCATCATAGGGCAGCTC |  |
| p50-F | AACAGAGAGGATTTCGTTTCCG | RT-qPCR |
| p50-R | TTTGACCTGAGGGTAAGACTTCT |  |
| BIRC2-F | GAATCTGGTTTCAGCTAGTCTGG | RT-qPCR |
| BIRC2-R | GGTGGGAGATAATGAATGTGCAA |  |
| BCL-2-F | CAGGATAACGGAGGCTGGGATG | RT-qPCR |
| BCL-2-R | AGAAATCAAACAGAGGCCGCA |  |
| CCL4-F | CTGTGCTGATCCCAGTGAATC | RT-qPCR |
| CCL4-R | TCAGTTCAGTTCCAGGTCATACA |  |
| TNF-α-F | GCCGCATCGCCGTCTCCTAC | RT-qPCR |
| TNF-α-R | CCTCAGCCCCCTCTGGGGTC |  |
| IL-6-F | ACTCACCTCTTCAGAACGAATTG | RT-qPCR |
| IL-6-R | CCATCTTTGGAAGGTTCAGGTTG |  |
| IL-8-F | ACTGAGAGTGATTGAGAGTGGAC | RT-qPCR |
| IL-8-R | AACCCTCTGCACCCAGTTTTC |  |
| IL-1β-F | ATGATGGCTTATTACAGTGGCAA | RT-qPCR |
| IL-1β-R | GTCGGAGATTCGTAGCTGGA |  |
| IFNα-F | TTGGCTGTGAAGAAATACTTCC | RT-qPCR |
| IFNα-R | GTTTGTTGATAAAGAGAGGGAT |  |
| IFNβ-F | ATGACCAACAAGTGTCTCCTCC | RT-qPCR |
| IFNβ-R | GGAATCCAAGCAAGTTGTAGCTC |  |
| ISG15-F | TCCTGGTGAGGAATAACAAGGG | RT-qPCR |
| ISG15-R | CTCAGCCAGAACAGGTCGTC |  |
| MXA-F | GGTGGTCCCCAGTAATGTGG | RT-qPCR |
| MXA-R | CGTCAAGATTCCGATGGTCCT |  |
| LTR-F | GGAGTACTACAAAGACTGCT | RT-PCR |
| LTR-R | TAACCAGAGAGACCCAGTA |  |
| siSER5 | Sense: CACCGTCTACATCTACTCCTA | siRNA |
| siMDA5 | Sense: AGAAUAACUCAUCAGAAUCTT | siRNA |
| siRIG-I | Sense: CAGAAUCUUAGUGAGAAUUTT | siRNA |
| siTRIM40-1 | Sense: GGAGAAAGGAGUCAGUGAA | siRNA |
| siTRIM40-2 | Sense: GAAGAAUGCUGGUGACUUA | siRNA |
| siTRIM40-3 | Sense: GGAGCAGGAAGCUCAGAAA | siRNA |
| sgSER5 | Sense: caccgCGACGACCAGAATGAAGTAG | sgRNA |
|  | Anti-sense: aaacCTACTTCATTCTGGTCGTCGc |  |
| sgNT | Sense: caccgCACCGAAGATGAAAGGAAAGGCGTT | sgRNA |
|  | Anti-sense: aaacAAACAACGCCTTTCCTTTCATCTTCc |  |
